# Methylnaltrexone Potentiates the Anti-Angiogenic Effects of mTOR Inhibitors

**DOI:** 10.1186/2040-2384-2-5

**Published:** 2010-02-19

**Authors:** Patrick A Singleton, Nurbek Mambetsariev, Frances E Lennon, Biji Mathew, Jessica H Siegler, Liliana Moreno-Vinasco, Ravi Salgia, Jonathan Moss, Joe GN Garcia

**Affiliations:** 1 Department of Medicine, University of Chicago, 5841 S. Maryland Avenue, W604, Chicago, IL, 60637, USA; 2 Department of Anesthesia and Critical Care, University of Chicago, 5841 S Maryland Avenue, MC 4028, Chicago, IL, 60637, USA

## Abstract

**Background:**

Recent cancer therapies include drugs that target both tumor growth and angiogenesis including mammalian target of rapamycin (mTOR) inhibitors. Since mTOR inhibitor therapy is associated with significant side effects, we examined potential agents that can reduce the therapeutic dose.

**Methods:**

Methylnaltrexone (MNTX), a peripheral mu opioid receptor (MOR) antagonist, in combination with the mTOR inhibitors temsirolimus and/or rapamycin, was evaluated for inhibition of VEGF-induced human pulmonary microvascular endothelial cell (EC) proliferation and migration as well as in vivo angiogenesis (mouse Matrigel plug assay).

**Results:**

MNTX inhibited VEGF-induced EC proliferation and migration with an IC50 of ~100 nM. Adding 10 nM MNTX to EC shifted the IC50 of temsirolimus inhibition of VEGF-induced proliferation and migration from ~10 nM to ~1 nM and from ~50 to ~10 nM respectively. We observed similar effects with rapamycin. On a mechanistic level, we observed that MNTX increased EC plasma membrane-associated tyrosine phosphate activity. Inhibition of tyrosine phosphatase activity (3,4-dephostatin) blocked the synergy between MNTX and temsirolimus and increased VEGF-induced tyrosine phosphorylation of Src with enhanced PI3 kinase and mTOR Complex 2-dependent phosphorylation of Akt and subsequent activation of mTOR Complex 1 (rapamycin and temsirolimus target), while silencing Src, Akt or mTOR complex 2 components blocked VEGF-induced angiogenic events.

**Conclusions:**

Our data indicate that MNTX exerts a synergistic effect with rapamycin and temsirolimus on inhibition of VEGF-induced human EC proliferation and migration and in vivo angiogenesis. Therefore, addition of MNTX could potentially lower the dose of mTOR inhibitors which could improve therapeutic index.

## Background

Recent therapeutic interventions for the inhibition of cancer progression include drugs that target both tumor growth and angiogenesis. Mammalian target of rapamycin (mTOR) inhibitors, including sirolimus (rapamycin) and temsirolimus, are potential therapeutic agents for hepatocellular cancer and renal cell carcinoma due to their anti-proliferative and anti-angiogenic properties. However, these mTOR inhibitors are often associated with unwanted side effects including rash, asthenia, mucositis, nausea, edema, anemia, hyperglycemia, thrombocytopenia, hyperlipaenia and anorexia [1-5]. Therefore, agents that can reduce the therapeutic concentration of these drugs could have significant clinical utility. We recently demonstrated that mu opioid agonists stimulate VEGF-induced angiogenesis via receptor transactivation and that mu opioid antagonists can inhibit VEGF receptor signaling [6]. During the course of these investigations, we also noted an effect of the peripheral opiate antagonist methylnaltrexone (MNTX) on endothelial cell migration and proliferation that occurred beyond the VEGF receptor, through a mechanism that involves inhibition of Src and Akt. We therefore hypothesized that methylnaltrexone could have synergistic effects with anti-angiogenic drugs (i.e. mTOR inhibitors).

In this study, we demonstrate that methylnaltrexone (MNTX) acts synergistically with the mTOR inhibitors, rapamycin and temsirolimus, on inhibition of VEGF-induced angiogenic events. Specifically, MNTX inhibited EC proliferation with an IC50 of ~100 nM. Adding 10 nM MNTX shifted the IC50 of temsirolimus on EC proliferation from ~10 nM to ~1 nM. Further, adding 10 nM MNTX shifted the IC50 of temsirolimus on inhibition of EC migration from ~50 nM to ~10 nM. The synergistic effects of MNTX and temsirolimus were also demonstrated in an in vivo model of angiogenesis (mouse Matrigel plug assay). There was a shift in the IC50 on inhibition of VEGF-induced EC proliferation and migration with MNTX and rapamycin. The synergistic mechanism involves MNTX activation of tyrosine phosphatase activity with consequent inhibition of VEGF-induced Src activation. MNTX-induced Src inactivation results in inhibition of PI3 kinase and mTOR signaling required for Akt activation (serine/threonine phosphorylation). These results suggest addition of MNTX could potentially lower the therapeutic doses of mTOR inhibitors including rapamycin and temsirolimus.

## Methods

### Cell Culture and Reagents

Human pulmonary microvascular EC (HPMVEC) were obtained from Cambrex (Walkersville, MD) and cultured as previously described [7,8] in EBM-2 complete medium (Cambrex) at 37°C in a humidified atmosphere of 5% CO_2_, 95% air, with passages 6-10 used for experimentation. Unless otherwise specified, reagents were obtained from Sigma (St. Louis, MO). Vascular endothelial growth factor (VEGF) was purchased from R&D Systems (Minneapolis, MN). Methylnaltrexone bromide or methylnaltrexone (MNTX) was purchased from Mallinckrodt Specialty Chemicals (Phillipsburg, NJ). Temsirolimus was acquired through Wyeth Pharmaceuticals. Rapamycin was purchased from Sigma (St. Louis, MO). Reagents for SDS-PAGE electrophoresis were purchased from Bio-Rad (Richmond, CA) and Immobilon-P transfer membrane was purchased from Millipore (Millipore Corp., Bedford, MA). Rabbit anti-pSer^473^Akt, rabbit anti-pThr^308^Akt, rabbit anti-Akt, rabbit anti-pThr^389 ^p70 S6K and anti-p70 S6K antibodies were purchased from Cell Signaling Technologies (Danvers, MA). Rabbit anti-mTOR, rabbit anti-Rictor and rabbit anti-FKBP12 antibodies were purchased from Santa Cruz Biotechnology (Santa Cruz, CA). Mouse anti-pp60src antibody was purchased from Upstate Biotechnologies (Lake Placid, NY). LY294002 was purchased from EMD Biosciences (Gibbstown, NJ). Mouse anti-β-actin antibody, rabbit anti-phospho-tyrosine^418 ^Src antibody and naltrexone, were purchased from Sigma (St. Louis, MO). Secondary horseradish peroxidase (HRP)-labeled antibodies were purchased from Amersham Biosciences (Piscataway, NJ).

### Immunoprecipitation and Immunoblotting

Cellular materials from treated or untreated HPMVEC were incubated with IP buffer (50 mM HEPES (pH 7.5), 150 mM NaCl, 20 mM MgCl_2_, 1% Nonidet P-40 (NP-40), 0.4 mM Na_3_VO_4_, 40 mM NaF, 50 μM okadaic acid, 0.2 mM phenylmethylsulfonyl fluoride, 1:250 dilution of Calbiochem protease inhibitor mixture 3). The samples were then immunoprecipitated with either anti-Raptor or anti-Rictor IgG followed by SDS-PAGE in 4-15% polyacrylamide gels, transfer onto Immobilon™ membranes, and developed with specific primary and secondary antibodies. Visualization of immunoreactive bands was achieved using enhanced chemiluminescence (Amersham Biosciences).

### Transfection of siRNA against mTOR, Src, Rictor, FKBP12 and Akt

The siRNA for human mTOR, Src, Rictor, FKBP12 and Akt were purchased from Santa Cruz Biotechnology (Santa Cruz, CA) and were utilized according to the manufacturer's specifications. Briefly, human lung EC were transfected with siRNA using siPORTamine™ as the transfection reagent (Ambion, TX). Cells (~40% confluent) were serum-starved for 1 hour followed by incubated with 250 nM of target siRNA (or scramble siRNA or no siRNA) for 6 hours in serum-free media. The serum-containing media was then added (1% serum final concentration) for 42 h before biochemical experiments and/or functional assays were conducted.

### Human Pulmonary Microvascular EC Migration Assay

Twenty-four transwell units with 8 μM pore size were used for monitoring *in vitro *cell migration. HPMVEC (~1 × 10^4 ^cells/well) were plated with various treatments (MNTX, temsirolimus, LY294002, 3,4-Dephostatin or siRNA) to the upper chamber and VEGF (100 nM) was added to the lower chamber. Cells were allowed to migrate for 18 hours. Cells from the upper and lower chamber were quantitated using the CellTiter96™ MTS assay (Promega, San Luis Obispo, CA) and read at 492 nm. % migration was defined as the # of cells in the lower chamber % the number of cells in both the upper and lower chamber. Each assay was set up in triplicate, repeated at least five times and analyzed statistically by Student's *t *test (with statistical significance set at *P *< 0.05).

### Human Pulmonary Microvascular EC Proliferation Assay

For measuring cell growth, HPMVEC [5 × 10^3 ^cells/well pretreated with various agents (MNTX, temsirolimus, LY294002, 3,4-Dephostatin or siRNA) were incubated with 0.2 ml of serum-free media containing 100 nM VEGF for 24 h at 37°C in 5%CO_2_/95% air in 96-well culture plates. The *in vitro *cell proliferation assay was analyzed by measuring increases in cell number using the CellTiter96™ MTS assay (Promega, San Luis Obispo, CA) and read at 492 nm. Each assay was set up in triplicate, repeated at least five times and analyzed statistically by Student's *t *test (with statistical significance set at *P *< 0.05).

### Tyrosine Phosphatase Assay

HLMVEC were treated with MNTX (100 nM), VEGF (100 nM) and/or morphine (100 nM) for 5 minutes. Lysates were then obtained and further purified to enrich plasma membrane-associated proteins by wheat germ agglutinin (WGA) affinity as previously described [9] and analyzed for tyrosine phosphatase activity using the fluorometric Rediplate™ 96 EnzChek Tyrosine Phosphatase Assay Kit (Invitrogen (Molecular Probes), Eugene, OR) as we have previously described [10]. Briefly, cellular materials are incubated in reaction buffer at 30°C and then added to a 96 well plate coated with 6,8-difluoro-4-methylumbelliferyl phosphate (DiFMUP). Tyrosine phosphatase activity cleaves DiFMUP into DiFMU with an excitation/emission maxima of 358/452 nm.

### In Vivo Angiogenesis Assay

The Matrigel plug assay was used to assess in vivo angiogenesis [11]. 10-week-old female C57BL/6 mice (Jackson Laboratory, Bar Harbor, ME) were injected subcutaneously on the ventral abdomen with 500 μl Matrigel (BD Biosciences, San Jose, CA) containing either MNTX (100 nM), temsirolimus (10 nM), or both drugs (100 nM MNTX and 10 nM temsirolimus). 20 ng VEGF was added to all Matrigel plugs. After 21 days, the plugs were removed and analyzed for hemoglobin content. The plugs were weighed and homogenized, and their hemoglobin content was quantified using the QuantiChrom™ hemoglobin assay kit (BioAssay Systems, Hayward, CA).

## Results

### Analysis of methylnaltrexone (MNTX) synergy with mTOR inhibitors on inhibition of human endothelial cell (EC) proliferation and migration

**Figure 1:**
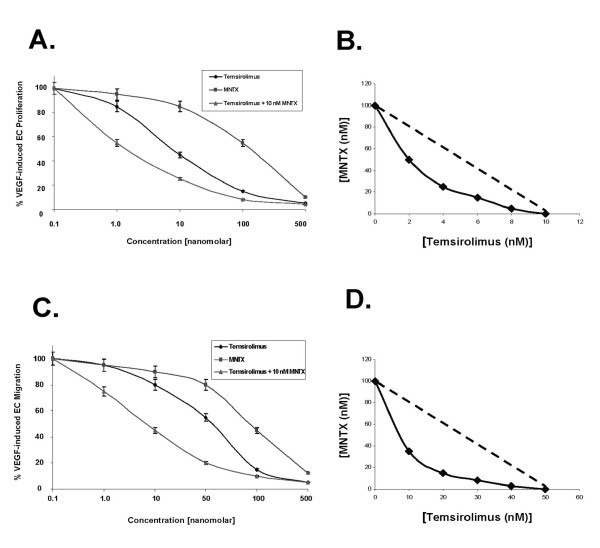
**Determination of methylnaltrexone (MNTX) synergistic effects with temsirolimus on inhibition of VEGF-induced human endothelial cell (EC) proliferation and migration**. **Panel A**: Graphical representation of human EC assayed for VEGF (100 nM)-induced proliferation (24 hours) in the presence or absence of 0.1, 1.0, 10, 100 or 500 nM MNTX, temsirolimus, or temsirolimus + 10 nM MNTX. Experiments were performed in triplicate. Error bars = standard deviation. **Panel B**: Isobologram analysis of the combination of MNTX and temsirolimus on inhibition of VEGF-induced proliferation. The dashed line indicates a zero interaction for the isobole. The shift to the left indicates a synergistic interaction. **Panel C**: Graphical representation of human EC assayed for VEGF (100 nM)-induced migration (24 hours) in the presence or absence of 0.1, 1.0, 10, 100 or 500 nM MNTX, temsirolimus, or temsirolimus + 10 nM MNTX. Experiments were performed in triplicate. Error bars = standard deviation. **Panel D**: Isobologram analysis of the combination of MNTX and temsirolimus on inhibition of VEGF-induced migration. The dashed line indicates a zero interaction for the isobole. The shift to the left indicates a synergistic interaction.

**Figure 2:**
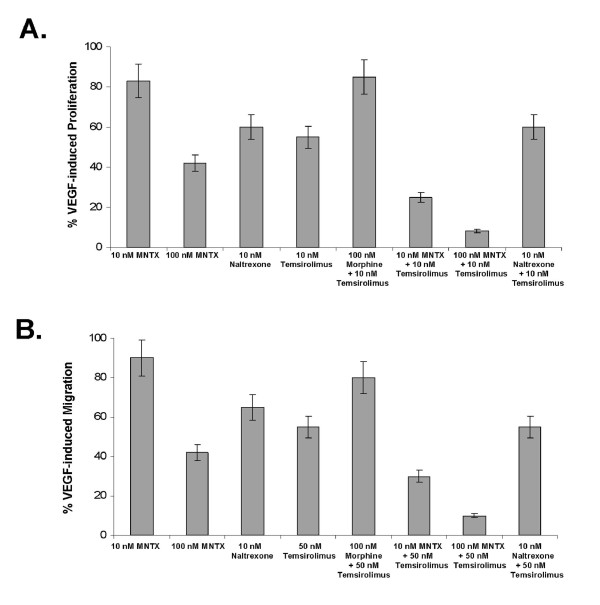
**Comparison of MNTX and naltrexone on synergy with temsirolimus on VEGF-induced angiogenic events**. **Panel A**: Bar graph representation of human EC assayed for VEGF (100 nM)-induced proliferation (24 hours) in the presence or absence of 10 nM MNTX, 100 nM MNTX, 10 nM naltrexone, 10 nM temsirolimus, 100 nM morphine + 10 nM temsirolimus, 10 nM MNTX + 10 nM temsirolimus, 100 nM MNTX + 10 nM temsirolimus or 10 nM naloxone + 10 nM temsirolimus. Experiments were performed in triplicate. Error bars = standard deviation. **Panel B**: Bar graph representation of human EC assayed for VEGF (100 nM)-induced migration (24 hours) in the presence or absence of 10 nM MNTX, 100 nM MNTX, 10 nM naltrexone, 10 nM temsirolimus, 100 nM morphine + 10 nM temsirolimus, 10 nM MNTX + 10 nM temsirolimus, 100 nM MNTX + 10 nM temsirolimus or 10 nM naloxone + 10 nM temsirolimus. Experiments were performed in triplicate. Error bars = standard deviation.

**Figure 3:**
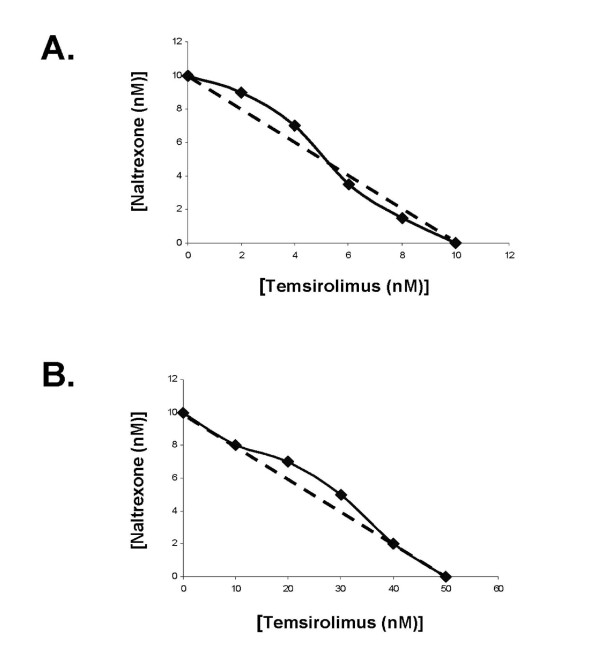
**Isobologram analysis indicating a lack of synergy of naltrexone with temsirolimus on VEGF-induced human EC proliferation and migration**. **Panel A**: Isobologram analysis of the combination of naltrexone and temsirolimus on inhibition of VEGF-induced proliferation. The dashed line indicates a zero interaction for the isobole. The close proximity of the data with the dashed line indicates there is not a synergistic interaction. **Panel B**: Isobologram analysis of the combination of naltrexone and temsirolimus on inhibition of VEGF-induced migration. The dashed line indicates a zero interaction for the isobole. The close proximity of the data with the dashed line indicates there is not a synergistic interaction.

Given our previous published data indicating that MNTX inhibits VEGF-induced Akt activation [10], we hypothesized that MNTX could have synergistic effects with anti-angiogenic drugs that regulate Akt signaling including mTOR inhibitors. Figure 1-A indicates that MNTX inhibits EC proliferation with an IC50 of ~100 nM. Adding ten fold lower concentration of MNTX (10 nM) to human EC shifted the IC50 of temsirolimus from ~10 nM to ~1 nM. These results were further confirmed with isobologram analysis [12] (Figure 1-B). Adding 10 nM MNTX shifted the IC50 of temsirolimus on inhibition of EC migration from ~50 nM to ~10 nM (Figure 1-C) and the synergy was confirmed using isobologram analysis [12] (Figure 1-D). These synergistic effects were not observed with the uncharged mu opioid antagonist, naltrexone (Figure 2-A, B; Figure 3-A, B). The synergistic effects of MNTX were paralleled with the mTOR inhibitor, rapamycin (see Additional Figure 1).

### The roles of mTOR Complex components, Akt and Src in MNTX and temsirolimus inhibition of VEGF-induced angiogenesis

**Figure 4:**
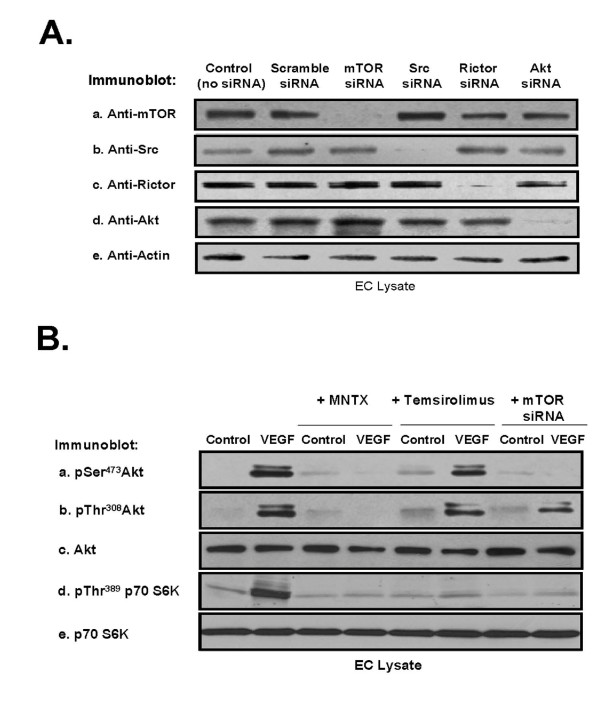
**Analysis of MNTX, temsirolimus and mTOR regulation of VEGF-induced Akt activation in human EC**. **Panel A**: Human EC were either untreated (control) or treated with scramble siRNA, mTOR siRNA, Src siRNA, Rictor siRNA or Akt siRNA for 48 hours, lysates obtained, run on SDS-PAGE and immunoblotted with anti-mTOR (a), anti-Src (b), anti-Rictor (c), anti-Akt (d) or anti-actin (e) antibody. **Panel B**: Human EC were serum starved for one hour and either untreated (control) or treated with VEGF (100 nM, 5 minutes) with or without pretreatment (1 hour) with 100 nM MNTX, 100 nM temsirolimus, or pretreatment for 48 hours with mTOR siRNA. EC lysates were obtained, run on SDS-PAGE and immunoblotted with anti-pSer^473^Akt (a), anti-pThr^308^Akt (b), anti-AKT (c), anti-pThr389 p70 S6K (d) or anti-p70 S6K (e) antibody.

We next examined the mechanism(s) of the synergistic effects of MNTX with temsirolimus on inhibition of VEGF-induced angiogenic events. Our previous published data indicate that Akt activation is important in VEGF-induced angiogenesis [10]. Akt is activated by threonine phosphorylation (T^308^) in the catalytic domain by PI3 kinase-dependent PDK-1 and by serine phosphorylation (S^473^) in the hydrophobic motif by various kinases including mTOR [13-15]. The substrate specificity of mTOR is regulated by complex formation with other proteins. Specifically, mTOR exists in a rapamycin-sensitive complex (mTOR Complex 1) with the regulatory-associated protein of mTOR (Raptor) and a rapamycin-insensitive complex (mTOR Complex 2) with the rapamycin-insensitive companion of mTOR, Rictor [1-5,13,15]. We silenced (siRNA) selective proteins in human EC including mTOR (Figure 4-A). Pre-treating human EC with MNTX, temsirolimus or mTOR siRNA followed by VEGF challenge revealed that Akt activation (serine/threonine phosphorylation) is blocked by MNTX. Further, silencing mTOR blocked VEGF-induced serine, but not threonine Akt phosphorylation. Interestingly, the mTOR inhibitor, temsirolimus, did not attenuate Akt activation but inhibited the mTOR Complex 1 target p70 S6K [4] (Figure 4-B).

**Figure 5:**
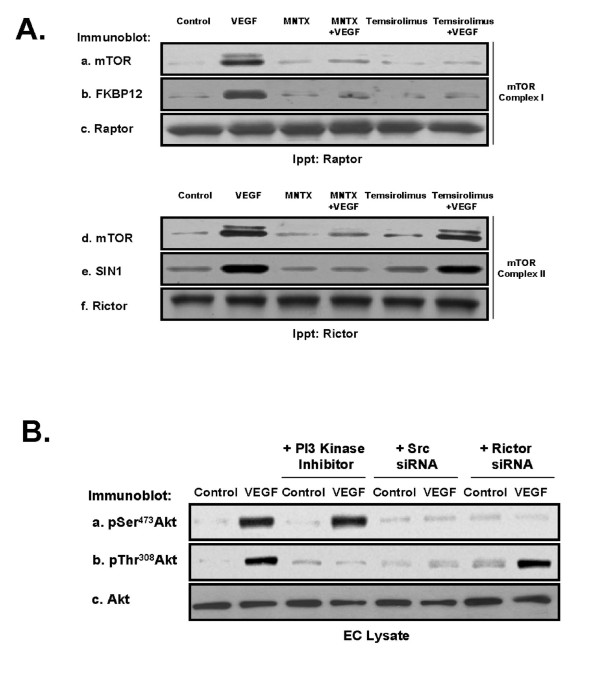
**Determination of mTOR Complex 1 and mTOR Complex 2 regulation of VEGF-induced Akt activation in human EC. Panel A**: Human EC were serum starved for one hour and either untreated (control) or treated with VEGF (100 nM, 5 minutes) with or without pretreatment (1 hour) with 100 nM MNTX or 100 nM temsirolimus. EC were then solublized in IP buffer (50 mM HEPES (pH 7.5), 150 mM NaCl, 20 mM MgCl_2_, 1% Nonidet P-40 (NP-40), 0.2 mM phenylmethylsulfonyl fluoride, 1:250 dilution of Calbiochem protease inhibitor mixture 3) and immunoprecipitated with either anti-Raptor (mTOR Complex 1 component) or anti-Rictor (mTOR Complex 2 component) antibody. The immunoprecipitated material was run on SDS-PAGE and immunoblotted with anti-mTOR (a, d), anti-FKBP12 (mTOR Complex 1 component and direct target of temsirolimus) (b), anti-Raptor (c), anti-SIN1 (mTOR Complex 2 component) (e) or anti-Rictor (f) antibody. **Panel B**: Human EC were serum starved for one hour and either untreated (control) or treated with VEGF (100 nM, 5 minutes) with or without pretreatment (1 hour) with LY294002 (PI3 kinase inhibitor, 10 μM) or pretreated for 48 hours with Src or Rictor (mTOR Complex 2 component) siRNA. EC lysates were obtained, run on SDS-PAGE and immunoblotted with anti-pSer^473^Akt (a), anti-pThr^308^Akt (b), or anti-AKT (c) antibody.

To further investigate the roles of MNTX and temsirolimus in VEGF-mediated Akt signaling, we examined two main mTOR-associated protein complexes, mTOR Complex 1, consisting of various proteins including mTOR, FKBP12 (a major target of mTOR inhibitors including temsirolimus) and Raptor, and mTOR Complex 2, consisting of various proteins including SIN1 and Rictor. Immunoprecipitation with either Rictor or Raptor antibody after VEGF treatment of human EC with MNTX or temsirolimus pre-treatment indicated that VEGF induces mTOR Complex 1 and mTOR Complex 2 formation. Both MNTX and temsirolimus block mTOR Complex 1 formation while only MNTX blocks mTOR Complex 2 formation (Figure 5-A).

We and others have previously published that VEGF induces Src and PI3 kinase activation in human EC [6,16,17]. We inhibited PI3 kinase activity with LY294002 or silenced (siRNA) Src or Rictor (required for mTOR Complex 2 formation), challenged EC with VEGF and examined Akt activation. Our results (Figure 5-B) indicate that Src is required for both serine and threonine phosphorylation of Akt, the PI3 kinase pathway is required for threonine phosphorylation of Akt and mTOR Complex 2 is required for serine phosphorylation of Akt.

**Figure 6:**
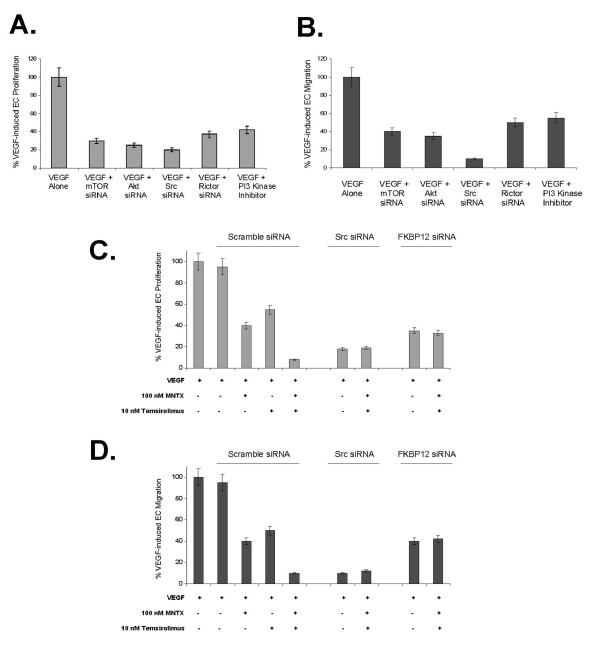
**The effects of mTOR, Akt, Src, Rictor and PI3 kinase on VEGF-induced human EC proliferation and migration**. **Panel A**: Bar graph representation of human EC assayed for VEGF (100 nM)-induced proliferation (24 hours) in the presence or absence of 48 hour pretreatment with mTOR siRNA, Akt siRNA, Src siRNA, Rictor (mTOR Complex 2 component) siRNA or pretreatment (1 hour) with LY294002 (PI3 kinase inhibitor, 10 μM). Experiments were performed in triplicate. Error bars = standard deviation. **Panel B**: Bar graph representation of human EC assayed for VEGF (100 nM)-induced migration (24 hours) in the presence or absence of 48 hour pretreatment with mTOR siRNA, Akt siRNA, Src siRNA, Rictor (mTOR Complex 2 component) siRNA or pretreatment (1 hour) with LY294002 (PI3 kinase inhibitor, 10 μM). Experiments were performed in triplicate. Error bars = standard deviation. **Panel C**: Bar graph representation of human EC assayed for VEGF (100 nM)-induced proliferation (24 hours) in the presence or absence of 48 hour pretreatment with Src or FKBP12 siRNA with addition of 100 nM MNTX and/or 10 nM temsirolimus. Experiments were performed in triplicate. Error bars = standard deviation. **Panel D**: Bar graph representation of human EC assayed for VEGF (100 nM)-induced migration (24 hours) in the presence or absence of 48 hour pretreatment with Src or FKBP12 siRNA with addition of 100 nM MNTX and/or 10 nM temsirolimus. Experiments were performed in triplicate. Error bars = standard deviation.

Similar to our results in Figures 4 and 5, we observed that silencing (siRNA) of mTOR, Akt, Src, Rictor or inhibition of PI3 kinase activity significantly attenuated VEGF-induced human EC proliferation (Figure 6-A) and migration (Figure 6-B) with Src silencing inducing the greatest inhibition of these activites. In addition, silencing Src or FKBP12 (direct target of temsirolimus [2,3,5]) blocked the synergy observed with MNTX and temsirolimus on VEGF-induced EC proliferation (Figure 6-C) and migration (Figure 6-D). However, our synergism analysis is complicated by the potent effects of Src and FKBP12 silencing alone.

### The role of tyrosine phosphatase activity in MNTX and temsirolimus inhibition of VEGF-mediated angiogenesis

**Figure 7:**
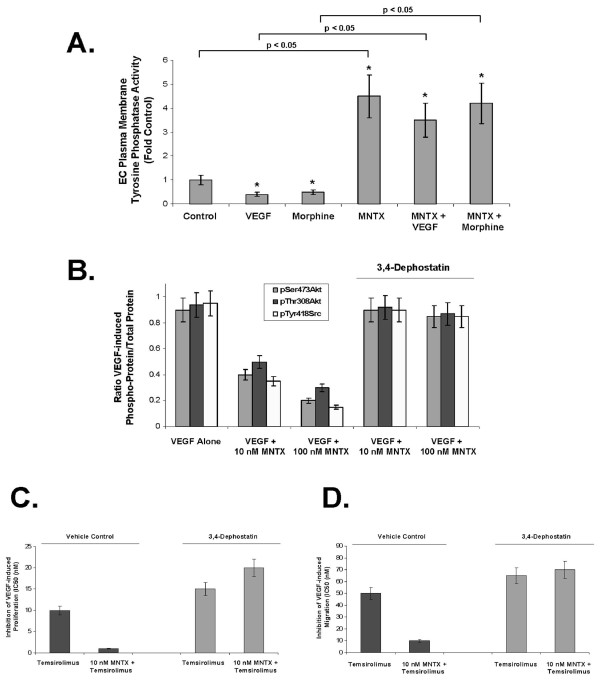
**3,4-Dephostatin inhibition of tyrosine phosphatase activity blocks MNTX synergistic effects with temsirolimus on inhibition of VEGF-induced angiogenic events**. **Panel A**: Bar graph representation of human EC plasma membrane-associated tyrosine phosphatase activity (see Methods) with VEGF (100 nM), morphine (100 nM), MNTX (100 nM) or combination treatment. The asterisks indicate a statistically significant difference (p < 0.05) between control and drug challenge. In addition, there is a statistically significant difference (p, 0.05) between without MNTX and with MNTX treatment. **Panel B**: Bar graph representation of the ration of phospho-protein to total protein using immunoblot analyses of pSer^473^Akt, pThr^308^Akt and pTyr^418^Src of human EC treated with 100 nM VEGF with or without 10 nM or 100 nM MNTX in the presence or absence of the tyrosine phosphatase inhibitor, 3,4-dephostatin (50 μM). Experiments were performed in triplicate. Error bars = standard deviation. **Panel C**: Graphical representation of the 50% inhibition concentration (IC50, (nM)) of human EC assayed for VEGF (100 nM)-induced proliferation (24 hours) of temsirolimus with or without 10 nM MNTX in the presence or absence of the tyrosine phosphatase inhibitor, 3,4-dephostatin (50 μM). Experiments were performed in triplicate. Error bars = standard deviation. **Panel D**: Graphical representation of the 50% inhibition concentration (IC50, (nM)) of human EC assayed for VEGF (100 nM)-induced migration (24 hours) of temsirolimus with or without 10 nM MNTX in the presence or absence of the tyrosine phosphatase inhibitor, 3,4-dephostatin (50 μM). Experiments were performed in triplicate. Error bars = standard deviation.

Our previous studies indicate that MNTX attenuates VEGF-induced pp60 Src activation (tyrosine phosphorylation) [6,10]. One possible mechanism of attenuating Src tyrosine phosphorylation is through tyrosine phosphatase activity [18,19]. To investigate this, we measured EC plasma membrane-associated tyrosine phosphatase activity and discovered that VEGF and morphine inhibit, while MNTX promotes tyrosine phosphatase activity (Figure 7-A). Treated of human EC with the potent tyrosine phosphatase inhibitor, 3.4-dephostatin [20] blocked MNTX inhibition of VEGF-induced Src and Akt activation (Figure 7-B) and reversed MNTX synergistic effects with temsirolimus on VEGF-induced proliferation (shift in the IC50 from ~1 nM to ~20 nM, Figure 7-C) and VEGF-induced migration (shift in the IC50 from ~5 nM to ~70 nM, Figure 7-D).

### In vivo analysis of MNTX synergy with temsirolimus on inhibition of angiogenesis

**Figure 8:**
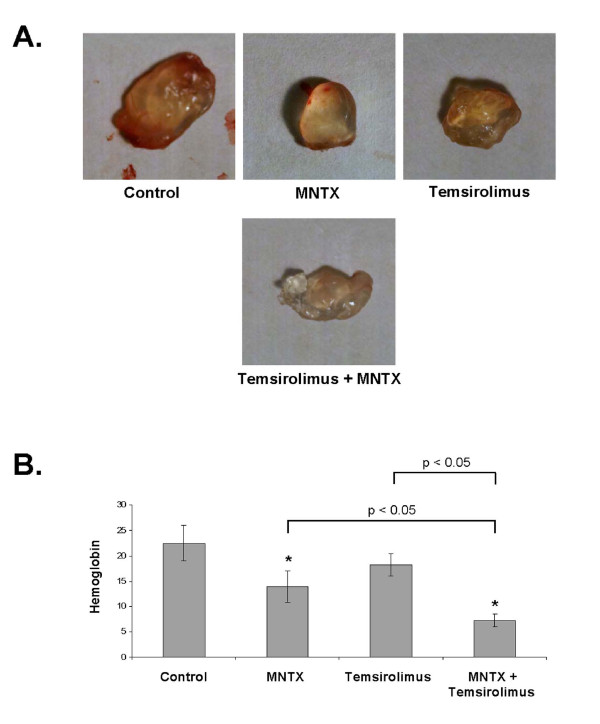
**In vivo analysis of MNTX synergy with temsirolimus on inhibition of angiogenesis**. In Panels A and B, 10-week-old female C57BL/6 mice (Jackson Laboratory, Bar Harbor, ME) were injected subcutaneously on the ventral abdomen with 500 μl Matrigel (BD Biosciences, San Jose, CA) containing either MNTX (100 nM), temsirolimus (10 nM), or both drugs (100 nM MNTX and 10 nM temsirolimus). 20 ng VEGF was added to all Matrigel plugs. After 21 days, the plugs were removed. **Panel A**: Pictures of Matrigel plugs after removal from mice. **Panel B**: Graphical analysis of Matrigel plug hemoglobin content. The plugs were weighed and homogenized, and their hemoglobin content was quantified using the QuantiChrom™ hemoglobin assay kit (BioAssay Systems, Hayward, CA), n = 3 per group. The asterisks indicate a statistically significant difference (p < 0.05) between treatment and control. There is also a statistically significant difference (p < 0.05) between MNTX or temsirolimus treatment alone versus in combination.

Considering the results of our in vitro human EC studies, we next examined the role of MNTX and temsirolimus on angiogenesis in vivo. In the mouse Matrigel plug assay [11], addition of 100 nM MNTX inhibited angiogenesis (Figure 8). Importantly, addition of MNTX in combination with temsirolimus inhibited angiogenesis to a greater extent than either drug alone (Figure 8). These results indicate MNTX and temsirolimus have a synergistic effect on inhibition of angiogenesis in vivo.

## Discussion

**Figure 9:**
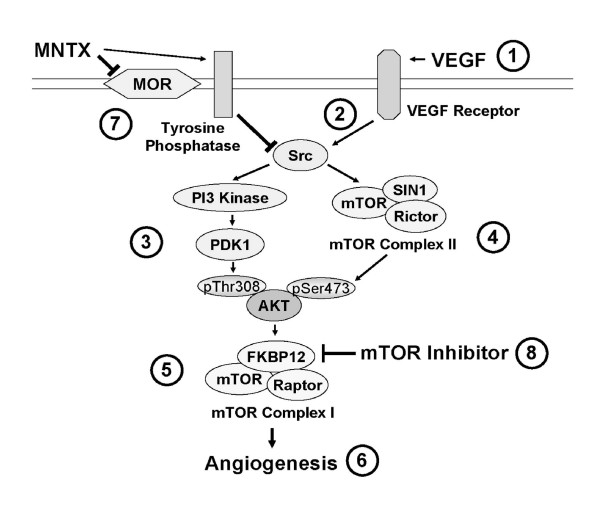
**Schematic diagram of a proposed mechanism of MNTX synergistic effects with temsirolimus on inhibition of VEGF-induced angiogenic events**. VEGF binding to VEGF receptors (1) induces Src activation (tyrosine phosphorylation)(2), Src-mediated PI3 kinase/PDK1 activation with consequent phospho-threonine^308 ^Akt phosphorylation (3) and mTOR Complex 2 formation (mTOR/SIN1/Rictor) with consequent phospho-serine^473 ^Akt phosphorylation (4). Activated (serine/threonine phosphorylated) Akt promotes mTOR Complex 1 formation (mTOR/FKBP12/Raptor) (5) required for EC proliferation and migration (6). MNTX inhibits the mu opioid receptor and promotes tyrosine phosphatase activity and Src inactivation (7). Temsirolimus binds to FKBP12 and inhibits mTOR Complex 2 formation (8). Therefore, the synergistic effects of MNTX with temsirolimus are achieved through inhibition of different components of a common VEGF-induced angiogenic signaling pathway. MNTX can have important clinical utility by potentially lower the therapeutic dose of temsirolimus in the treatment of various diseases requiring angiogenesis including cancer.

We and others have previously noted an effect of opiates on endothelial cell migration and proliferation, and an effect of opiate antagonists in attenuating opiate induced angiogenesis [6,8,10]. The selective peripheral antagonist of the mu opioid receptor, MNTX, administered subcutaneously, is approved in the USA, EU, Canada and Australia. In the USA, it is indicated for the treatment of opioid-induced constipation in patients with advanced illness (i.e. cancer, AIDS) who are receiving palliative care, when responses to laxatives have not been sufficient [21-24]. Use in attenuating other side effects of opiates has been studied [25]. In this study, we present the novel findings that MNTX acts in a synergistic manner with the mTOR inhibitors, rapamycin and temsirolimus, in inhibiting VEGF-induced angiogenic events. Our results indicate that the synergistic effects of MNTX with mTOR inhibitors are achieved through inhibition of different components of a common VEGF-induced angiogenic signaling pathway. MNTX inhibits the mu opioid receptor and stimulates tyrosine phosphatase activity which inhibits VEGF-induced Src activation and Src-regulated PI3 kinase and mTOR Complex 2-mediated Akt activation. Temsirolimus and rapamycin inhibit the downstream target of activated Akt, mTOR Complex 1 [1,15,26]. Inhibition of these events promotes synergistic inhibition of VEGF-induced angiogenesis (Figure 9). Therefore, we hypothesize that, in addition to its effects on GI motility, MNTX might have clinical utility by potentially lowering the therapeutic doses of mTOR inhibitors in the treatment of various diseases requiring angiogenesis including cancer.

We have focused our studies on methylnaltrexone (MNTX) because it is more likely to be used in advanced illness clinical settings than tertiary mu opioid receptor antagonists. Uncharged mu opioid antagonists, including naloxone and naltrexone, are fairly lipid soluble and cross the blood-brain barrier easily [27-29]. Despite numerous attempts at regulating doses, mu opioid antagonists have proven unsuitable for patients receiving opiates for pain management because of analgesia reversal and breakthrough pain [30]. MNTX is a quaternary derivative of the tertiary mu opiate antagonist naltrexone [31]. The addition of the methyl group to naltrexone at the amine in the ring forms the compound N-methylnaltrexone with greater polarity and lower lipid solubility. Because MNTX does not cross the blood-brain barrier, it could play a therapeutic role in reversing the peripheral effects of opiates in palliative care, especially for patients taking high doses of opiates for analgesia [32-37]. The plasma concentrations of morphine and MNTX in patients after parenteral or oral administration are consistent with the levels that regulated synergistic inhibition of VEGF-induced angiogenesis and inhibited Src in our *in vitro *model (IC50 = ~10 nM) [10].

We focused our studies on temsirolimus and rapamycin based on our previous published data that MNTX regulates VEGF-induced Akt activation [10] and the intricate relationship between Akt and mTOR pathways [5,26]. Both rapamycin and temsirolimus, a soluble ester analog of rapamycin, exert their action by binding to the intracellular protein, FKBP12, and inhibiting mTOR Complex 1 formation [38-40]. However, mTOR can still complex with SIN1 and Rictor (mTOR Complex 2) [1,15,26]. The mTOR Complex 2 serine phosphorylates Akt and is involved in actin cytoskeletal regulation [2,4,14,41,42]. Akt can also be threonine phosphorylated by PI3 kinase activation of PDK1 [41-45]. Activated (serine/threonine phosphorylated) Akt promotes mTOR Complex 1 assembly through inactivation (phosphorylation) of TSC2 and PRAS40 [5,26]. Activated mTOR Complex 1 phosphorylates several target proteins including S6K and 4EBP1 involved in cell proliferation, growth and survival [1,2,4,5,26].

The effects of MNTX on inhibition of mTOR described in this manuscript go beyond VEGF receptor activation and extend to downstream signaling pathways. We and others have previously reported that inhibition of Src protects from EC barrier disruption and angiogenesis [6,8,10]. Src regulates several potential angiogenic events including EC contraction and vascular permeability [43,44]. We extended these finding by observing that Src regulates VEGF-induced, PI3 kinase and mTOR-dependent, serine/threonine phosphorylation of Akt important for EC proliferation and migration. Further, Src regulates the synergistic effects of MNTX with temsirolimus on inhibition of VEGF-induced angiogenic events. We have previously demonstrated that MNTX increases tyrosine phosphatase activity, including RPTPμ [10]. This study extends these finding by demonstrating that the potent protein tyrosine phosphatase inhibitor, 3,4-Dephostatin, blocks MNTX inhibition of VEGF-induced Src and Akt phosphorylation. 3,4-Dephostatin is known to block the phosphatase activity of PTP-1B, SHPTP-1 and CD45 [20,40,46-48]. In addition, 3,4-Dephostatin increased insulin-induced tyrosine phosphorylation of PLCγ, c-Cbl and the regulatory subunit of PI3 kinase (a known Src substrate) [48]. We are currently examining the role of these tyrosine phosphatases in MNTX inhibition of VEGF-induced Src activation and angiogenesis.

Temsirolimus was approved by the FDA in 2007 for the treatment of advanced renal cell carcinoma, a disease resistant to existing chemotherapies (IFN-α, IL-2) [3,38-40]. There have been other attempts to potentiate the action of temsirolimus. In Phase 3 clinical trails, temsirolimus, IFN-α or temsirolimus + IFN-α treatment resulted in median survival rates of 10.9 months, 7.3 months and 8.4 months, respectively [3,38-40]. IFN-α did not augment temsirolimus treatment alone [3,38-40]. The results of these clinical trials indicate the need for an effective drug in temsirolimus combination therapy. Our observations that MNTX acts synergistically with mTOR inhibitors on inhibition of VEGF-induced angiogenic events merit clinical studies.

## Conclusions

Our results indicate that MNTX exerts a synergistic effect with rapamycin and temsirolimus on inhibition of VEGF-induced human EC proliferation and migration and in vivo angiogenesis. These synergistic effects are achieved through inhibition of different components of a common VEGF-induced angiogenic signaling pathway. MNTX inhibits the mu opioid receptor and stimulates tyrosine phosphatase activity which inhibits VEGF-induced Src activation and Src-regulated PI3 kinase and mTOR Complex 2-mediated Akt activation. Temsirolimus and rapamycin inhibit the downstream target of activated Akt, mTOR Complex 1. Inhibition of these events promotes synergistic inhibition of VEGF-induced angiogenesis (Figure 9). Therefore, addition of MNTX could potentially lower the dose of mTOR inhibitors which could improve therapeutic index.

## Competing interests

Dr. Moss serves as a paid consultant to Progenics Pharmaceuticals, Inc., has a financial interest in methylnaltrexone as a patent holder through the University of Chicago, and receives stock options from Progenics. Drs. Singleton and Garcia are co-inventors on patents with Dr. Moss. The other authors declare they have no competing interests.

## Authors' contributions

PAS helped conceive of the study, and participated in its design and coordination, performed the in vitro inhibition and isobologram assays and helped draft the manuscript. NM participated in the study design and conducted immunoprecipitation and immunoblot assays. FEL, BM and JHS participated in the study design and performed the in vivo Matrigel plug assays. LMV coordinated the animal studies by FEL, BM and JHS. RS participated in the design and coordination of the studies. JM helped conceive of the study, and participated in its design and coordination, and helped draft the manuscript. JGNG coordinated the work of LMV, BM and JHS. All authors read and approve the final manuscript.

## Supplementary Material

Additional file 1 **Determination of methylnaltrexone (MNTX) synergistic effects with rapamycin on inhibition of VEGF-induced human endothelial cell (EC) proliferation and migration**. This file shows through inhibition curves and isobologram analysis that MNTX acts synergistically with the mTOR inhibitor, rapamycin, on inhibition of VEGF-induced angiogenic events. Click here for file

## References

[B1] Brachmann S Fritsch C Maira SM Garcia-Echeverria C PI3K and mTOR inhibitors: a new generation of targeted anticancer agents Curr Opin Cell Biol 2009 21 194 8 10.1016/j.ceb.2008.12.011 19201591

[B2] Fasolo A Sessa C mTOR inhibitors in the treatment of cancer Expert Opin Investig Drugs 2008 17 1717 34 10.1517/13543784.17.11.1717 18922108

[B3] Malizzia LJ Hsu A Temsirolimus, an mTOR inhibitor for treatment of patients with advanced renal cell carcinoma Clin J Oncol Nurs 2008 12 639 46 10.1188/08.CJON.639-646 18676330

[B4] Abraham RT Gibbons JJ The mammalian target of rapamycin signaling pathway: twists and turns in the road to cancer therapy Clin Cancer Res 2007 13 3109 14 10.1158/1078-0432.CCR-06-2798 17545512

[B5] Faivre S Kroemer G Raymond E Current development of mTOR inhibitors as anticancer agents Nat Rev Drug Discov 2006 5 671 88 10.1038/nrd2062 16883305

[B6] Singleton PA Lingen MW Fekete MJ Garcia JG Moss J Methylnaltrexone inhibits opiate and VEGF-induced angiogenesis: Role of receptor transactivation Microvasc Res 2006 72 3 11 10.1016/j.mvr.2006.04.004 16820176

[B7] Singleton PA Dudek SM Ma SF Garcia JGN Transactivation of sphingosine 1-phosphate receptors is essential for vascular barrier regulation. Novel role for hyaluronan and CD44 receptor family J Biol Chem 2006 281 34381 93 10.1074/jbc.M603680200 16963454

[B8] Singleton PA Moreno-Vinasco L Sammani S Wanderling SL Moss J Garcia JGN Attenuation of Vascular Permeability by Methylnaltrexone: Role of mOP-R and S1P3 Transactivation Am J Respir Cell Mol Biol 2007 37 222 31 10.1165/rcmb.2006-0327OC 17395891

[B9] Wang Y Ao X Vuong H Konanur M Miller FR Goodison S Lubman DM Membrane Glycoproteins Associated with Breast Tumor Cell Progression Identified by a Lectin Affinity Approach J Proteome Res 2008 7 4313 4325 10.1021/pr8002547 18729497 PMC2630886

[B10] Singleton PA Garcia JGN Moss J Synergistic effects of methylnaltrexone with 5-fluorouracil and bevacizumab on inhibition of vascular endothelial growth factor-induced angiogenesis Mol Cancer Ther 2008 7 1669 79 10.1158/1535-7163.MCT-07-2217 18566238

[B11] Liu Z Kobayashi K van Dinther M van Heiningen SH Valdimarsdottir G van Laar T Scharpfenecker M Lowik CW Goumans MJ Ten Dijke P Pardali E VEGF and inhibitors of TGFbeta type-I receptor kinase synergistically promote blood-vessel formation by inducing alpha5-integrin expression J Cell Sci 2009 122 3294 302 10.1242/jcs.048942 19706683

[B12] Tallarida RJ Drug synergism: its detection and applications J Pharmacol Exp Ther 2001 298 865 72 11504778

[B13] Hutson TE Targeted therapy for renal cell carcinoma: a new treatment paradigm Proc (Bayl Univ Med Cent) 2007 20 244 8 17637878 10.1080/08998280.2007.11928297 PMC1906573

[B14] Jacinto E Loewith R Schmidt A Lin S Ruegg MA Hall A Hall MN Mammalian TOR complex 2 controls the actin cytoskeleton and is rapamycin insensitive Nat Cell Biol 2004 6 1122 8 10.1038/ncb1183 15467718

[B15] Sarbassov DD Ali SM Kim DH Guertin DA Latek RR Erdjument-Bromage H Tempst P Sabatini DM Rictor, a novel binding partner of mTOR, defines a rapamycin-insensitive and raptor-independent pathway that regulates the cytoskeleton Curr Biol 2004 14 1296 302 10.1016/j.cub.2004.06.054 15268862

[B16] Duval M Le Boeuf F Huot J Gratton JP Src-mediated phosphorylation of Hsp90 in response to vascular endothelial growth factor (VEGF) is required for VEGF receptor-2 signaling to endothelial NO synthase Mol Biol Cell 2007 18 4659 68 10.1091/mbc.E07-05-0467 17855507 PMC2043550

[B17] Dawson NS Zawieja DC Wu MH Granger HJ Signaling pathways mediating VEGF165-induced calcium transients and membrane depolarization in human endothelial cells Faseb J 2006 20 991 3 10.1096/fj.05-3923fje 16581961

[B18] den Hertog J Groen A Wijk T van der Redox regulation of protein-tyrosine phosphatases Arch Biochem Biophys 2005 434 11 5 10.1016/j.abb.2004.05.024 15629103

[B19] Stoker AW Protein tyrosine phosphatases and signalling J Endocrinol 2005 185 19 33 10.1677/joe.1.06069 15817824

[B20] Umezawa K Kawakami M Watanabe T Molecular design and biological activities of protein-tyrosine phosphatase inhibitors Pharmacol Ther 2003 99 15 24 10.1016/S0163-7258(03)00050-0 12804696

[B21] Slatkin N Thomas J Lipman AG Wilson G Boatwright ML Wellman C Zhukovsky DS Stephenson R Portenoy R Stambler N Israel R Methylnaltrexone for treatment of opioid-induced constipation in advanced illness patients J Support Oncol 2009 7 39 46 19278178

[B22] Thomas J Karver S Cooney GA Chamberlain BH Watt CK Slatkin NE Stambler N Kremer AB Israel RJ Methylnaltrexone for opioid-induced constipation in advanced illness N Engl J Med 2008 358 2332 43 10.1056/NEJMoa0707377 18509120

[B23] Portenoy RK Thomas J Moehl Boatwright ML Tran D Galasso FL Stambler N Von Gunten CF Israel RJ Subcutaneous methylnaltrexone for the treatment of opioid-induced constipation in patients with advanced illness: a double-blind, randomized, parallel group, dose-ranging study J Pain Symptom Manage 2008 35 458 68 10.1016/j.jpainsymman.2007.12.005 18440447

[B24] Methylnaltrexone (Relistor) for opioid induced constipation Med Lett Drugs Ther 2008 50 63 4 18688204

[B25] Moss J Rosow CE Development of peripheral opioid antagonists' new insights into opioid effects Mayo Clin Proc 2008 83 1116 30 10.4065/83.10.1116 18828971

[B26] Garcia JA Danielpour D Mammalian target of rapamycin inhibition as a therapeutic strategy in the management of urologic malignancies Mol Cancer Ther 2008 7 1347 54 10.1158/1535-7163.MCT-07-2408 18566209 PMC2587303

[B27] Moss J Foss J Pain Relief without Side Effects: Peripheral Opiate Antagonists 2005 Philadelphia: Lippincott Williams & Wilkins

[B28] Hoskin PJ Hanks GW Opioid agonist-antagonist drugs in acute and chronic pain states Drugs 1991 41 326 44 10.2165/00003495-199141030-00002 1711441

[B29] Greenwood-Van Meerveld B Gardner CJ Little PJ Hicks GA Dehaven-Hudkins DL Preclinical studies of opioids and opioid antagonists on gastrointestinal function Neurogastroenterol Motil 2004 16 Suppl 2 46 53 10.1111/j.1743-3150.2004.00555.x 15357851

[B30] Sykes NP Using Oral Naloxone in Management of Opioid Bowel Dysfunction 2005 New York: Haworth Medical Press

[B31] Yuan CS Foss JF O'Connor M Osinski J Karrison T Moss J Roizen MF Methylnaltrexone for reversal of constipation due to chronic methadone use: a randomized controlled trial Jama 2000 283 367 72 10.1001/jama.283.3.367 10647800

[B32] Stanski DR Greenblatt DJ Lowenstein E Kinetics of intravenous and intramuscular morphine Clin Pharmacol Ther 1978 24 52 9 657720 10.1002/cpt197824152

[B33] Neumann PB Henriksen H Grosman N Christensen CB Plasma morphine concentrations during chronic oral administration in patients with cancer pain Pain 1982 13 247 52 10.1016/0304-3959(82)90014-8 7122112

[B34] Collins SL Faura CC Moore RA McQuay HJ Peak plasma concentrations after oral morphine: a systematic review J Pain Symptom Manage 1998 16 388 402 10.1016/S0885-3924(98)00094-3 9879164

[B35] Yuan CS Doshan H Charney MR O'Connor M Karrison T Maleckar SA Israel RJ Moss J Tolerability, gut effects, and pharmacokinetics of methylnaltrexone following repeated intravenous administration in humans J Clin Pharmacol 2005 45 538 46 10.1177/0091270004273491 15831777

[B36] Cannom RR Mason RJ Methylnaltrexone: the answer to opioid-induced constipation? Expert Opin Pharmacother 2009 10 1039 45 10.1517/14656560902833914 19364251

[B37] Laustsen G Carrillo F Johnson J Smith C Drug approvals: '08 in review. Methylnaltrexone (Relistor) Nurse Pract 2009 34 31 10.1097/01.NPR.0000345266.90547.be 19155882

[B38] Bhatia S Thompson JA Temsirolimus in patients with advanced renal cell carcinoma: an overview Adv Ther 2009 26 55 67 10.1007/s12325-008-0138-3 19172239

[B39] Hudes G Carducci M Tomczak P Dutcher J Figlin R Kapoor A Staroslawska E Sosman J McDermott D Bodrogi I Kovacevic Z Lesovoy V Schmidt-Wolf IG Barbarash O Gokmen E O'Toole T Lustgarten S Moore L Motzer RJ Temsirolimus, interferon alfa, or both for advanced renal-cell carcinoma N Engl J Med 2007 356 2271 81 10.1056/NEJMoa066838 17538086

[B40] Figlin RA Mechanisms of Disease: survival benefit of temsirolimus validates a role for mTOR in the management of advanced RCC Nat Clin Pract Oncol 2008 5 601 9 10.1038/ncponc1173 18607393

[B41] Manning BD Cantley LC AKT/PKB signaling: navigating downstream Cell 2007 129 1261 74 10.1016/j.cell.2007.06.009 17604717 PMC2756685

[B42] Frost RA Lang CH Protein kinase B/Akt: a nexus of growth factor and cytokine signaling in determining muscle mass J Appl Physiol 2007 103 378 87 10.1152/japplphysiol.00089.2007 17332274

[B43] Mucha DR Myers CL Schaeffer RC Jr Endothelial contraction and monolayer hyperpermeability are regulated by Src kinase Am J Physiol Heart Circ Physiol 2003 284 H994 H1002 12456392 10.1152/ajpheart.00862.2002

[B44] Weis S Cui J Barnes L Cheresh D Endothelial barrier disruption by VEGF-mediated Src activity potentiates tumor cell extravasation and metastasis J Cell Biol 2004 167 223 9 10.1083/jcb.200408130 15504909 PMC2172541

[B45] Chen R Kim O Yang J Sato K Eisenmann KM McCarthy J Chen H Qiu Y Regulation of Akt/PKB activation by tyrosine phosphorylation J Biol Chem 2001 276 31858 62 10.1074/jbc.C100271200 11445557

[B46] Liu G Protein tyrosine phosphatase 1B inhibition: opportunities and challenges Curr Med Chem 2003 10 1407 21 10.2174/0929867033457296 12871138

[B47] Kakeya H Imoto M Takahashi Y Naganawa H Takeuchi T Umezawa K Dephostatin, a novel protein tyrosine phosphatase inhibitor produced by Streptomyces. II. Structure determination J Antibiot (Tokyo) 1993 46 1716 9 8270494 10.7164/antibiotics.46.1716

[B48] Suzuki T Hiroki A Watanabe T Yamashita T Takei I Umezawa K Potentiation of insulin-related signal transduction by a novel protein-tyrosine phosphatase inhibitor, Et-3,4-dephostatin, on cultured 3T3-L1 adipocytes J Biol Chem 2001 276 27511 8 10.1074/jbc.M011726200 11342532

