# Supplementary material for: Methylnaltrexone Potentiates the Anti-Angiogenic Effects of mTOR Inhibitors
Source: J Angiogenes Res. 2010 Feb 19;2:5. doi: 10.1186/2040-2384-2-5 (PMC2831839; doi:10.1186/2040-2384-2-5)
Supplement: Additional file 1 — Determination of methylnaltrexone (MNTX) synergistic effects with rapamycin on inhibition of VEGF-induced human endothelial cell (EC) proliferation and migration. This file shows through inhibition curves and isobologram analysis that MNTX acts synergistically with the mTOR inhibitor, rapamycin, on inhibition of VEGF-induced angiogenic events. [file 2040-2384-2-5-S1.PDF]

Additional File 1 -

1.

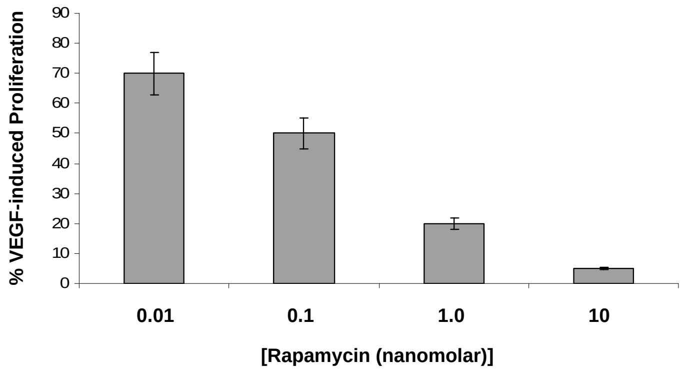

2.

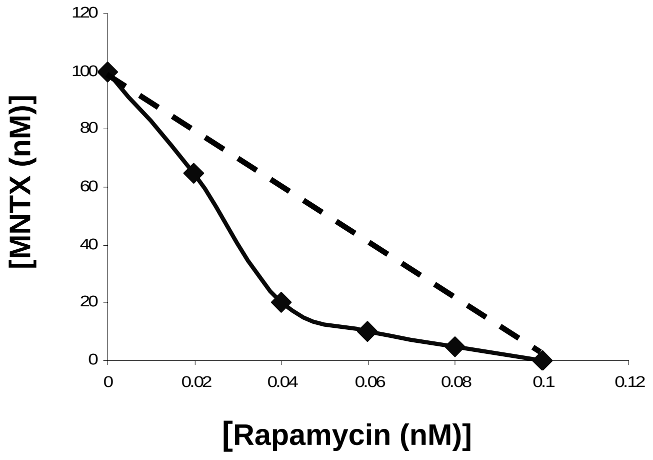

3.

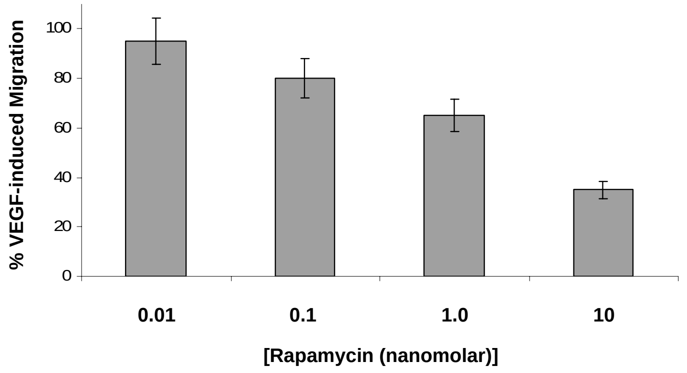

4.

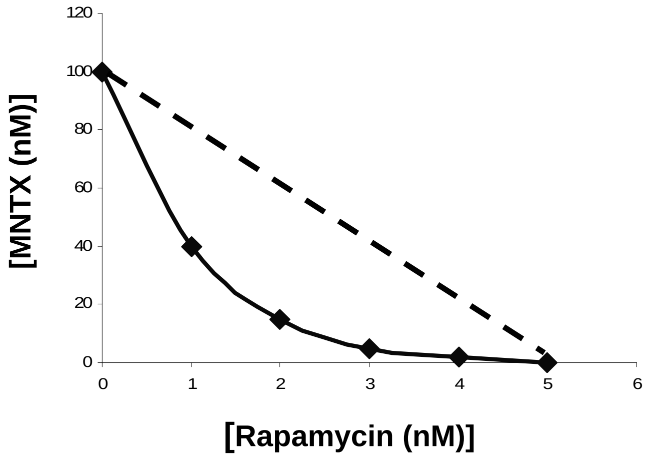

Additional File 1 continued –

5.

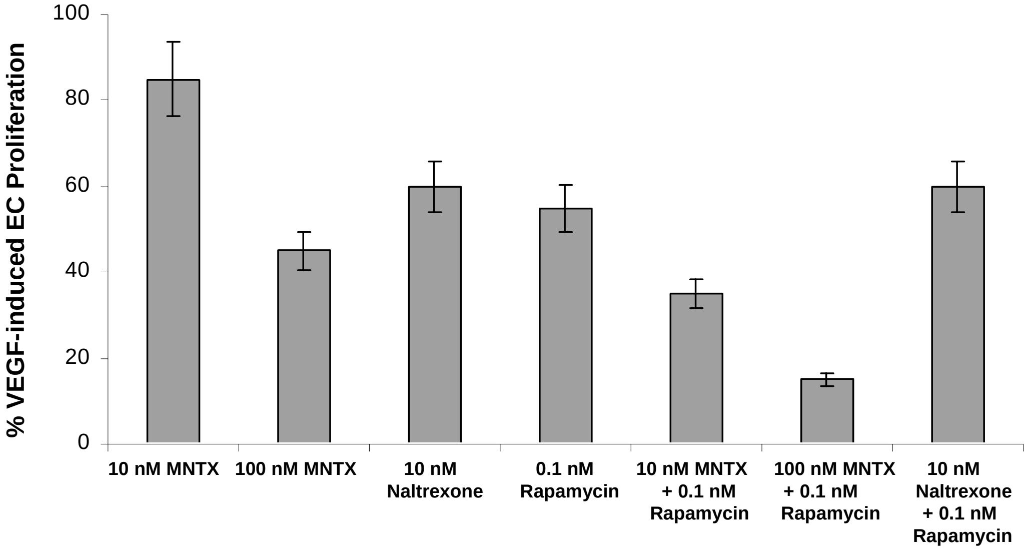

6.

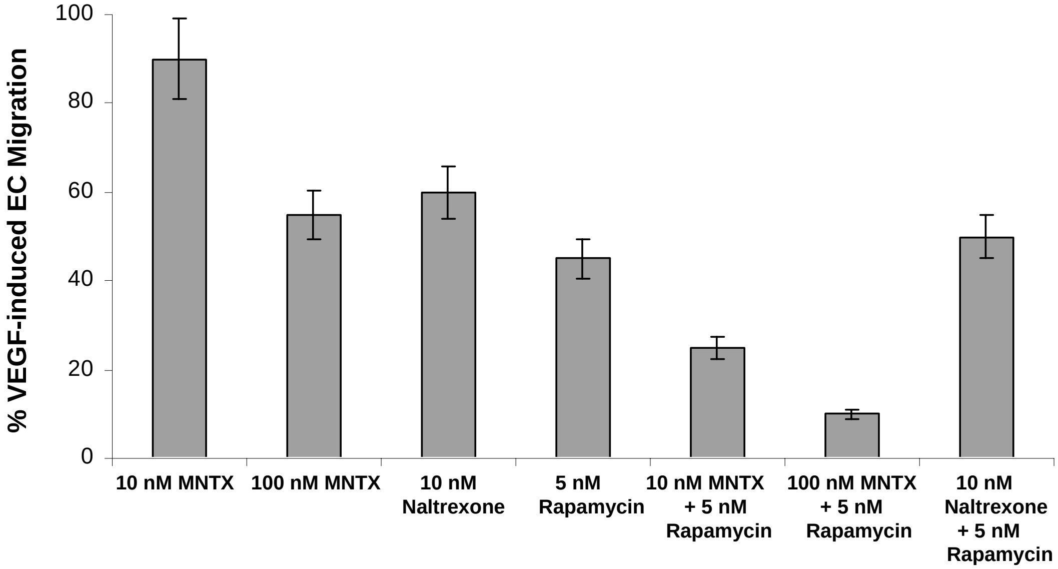

Additional File 1 – Determination of methylnaltrexone (MNTX) synergistic effects with rapamycin on inhibition of VEGF-induced human endothelial cell (EC) proliferation and migration. Panel 1: Bar graph representation of human EC assayed for VEGF (100 nM)-induced profileration (24 hours) in the presence or absence of 0.01,

0.1, 1.0 or 10 nM rapamycin. Experiments were performed in triplicate. Error bars = standard deviation. **Panel 2:** Isobologram analysis of the combination of MNTX and rapamycin on inhibition of VEGF-induced proliferation. The dashed line indicates a zero interaction for the isobole. The shift to the left indicates a synergistic interaction. **Panel 3:** Bar graph representation of human EC assayed for VEGF (100 nM)-induced migration (24 hours) in the presence or absence of 0.01, 0.1, 1.0 or 10 nM rapamycin. Experiments were performed in triplicate. Error bars = standard deviation. **Panel 4:** Isobologram analysis of the combination of MNTX and rapamycin on inhibition of VEGF-induced proliferation. The dashed line indicates a zero interaction for the isobole. The shift to the left indicates a synergistic interaction. **Panel 5:** Bar graph representation of human EC assayed for VEGF (100 nM)-induced profileration (24 hours) in the presence or absence of 10 nM MNTX, 100 nM MNTX, 10 nM naltrexone, 0.1 nM rapamycin, 10 nM MNTX + 0.1 nM rapamycin, 100 nM MNTX + 0.1 nM rapamycin or 10 nM naloxone + 0.1 nM rapamycin. Experiments were performed in triplicate. Error bars = standard deviation. **Panel 6:** Bar graph representation of human EC assayed for VEGF (100 nM)-induced migration (24 hours) in the presence or absence of 10 nM MNTX, 100 nM MNTX, 10 nM naltrexone, 5 nM rapamycin, 10 nM MNTX + 5 nM rapamycin, 100 nM MNTX + 5 nM rapamycin or 10 nM naloxone + 5 nM rapamycin. Experiments were performed in triplicate. Error bars = standard deviation.
